# Finite Element Simulation of Biomechanical Effects on Periodontal Ligaments During Maxillary Arch Expansion with Thermoformed Aligners

**DOI:** 10.3390/jfb16040143

**Published:** 2025-04-17

**Authors:** Gustavo A. Rojas, Jose Isidro García-Melo, Juan S. Aristizábal

**Affiliations:** 1 Mechanical Engineering Programm, Marco Fidel Suarez Military School (EMAVI), Cali 760001, Colombia; gustavo.rojasro@emavi.edu.co; 2 Engineering Faculty, Mechanical Engineering School, Universidad del Valle, Cali 760031, Colombia; juan.mulett@correounivalle.edu.co

**Keywords:** amelocemental junction, FEM simulation, maxillary arch, thermoformed aligners, complementary biomechanical attachments (CBAs)

## Abstract

Purpose: This paper investigates the biomechanical effect of thermoformed aligners equipped with complementary biomechanical attachments (CBAs) on periodontal ligaments (PDLs) during the expansion process of the maxillary arch. The analysis was conducted using advanced simulations based on the finite element method (FEM). Methods: High-resolution 3D CAD models were created for four tooth types: canine, first premolar, second premolar, and first molar. Additional 3D models were developed for aligners, CBAs, and PDLs. These were integrated into a comprehensive FEM model to simulate clinical rehabilitation scenarios. Validation was achieved through comparative analysis with empirical medical data. Results: The FEM simulations revealed the following: for canine, the displacement was 0.134 mm with a maximum stress of 4.822 KPa in the amelocemental junction. For the first premolar, the displacement was 0.132 mm at a maximum stress of 3.273 KPa in the amelocemental junction. The second premolar had a displacement of 0.129 mm and a stress of 1.358 KPa at 1 mm from the amelocemental junction; and first molar had a displacement of 0.124 mm and a maximum stress of 2.440 KPa. Conclusions: The inclusion of CBAs significantly reduced tooth tipping during maxillary arch expansion. Among the models tested, the vestibular CBA demonstrated superior performance, delivering optimal tooth movement when combined with thermoformed aligners. Significance: FEM techniques provide a robust and cost-effective alternative to in vivo experimentation, offering precise and reliable insights into the biomechanical efficacy of CBAs in thermoformed aligners. This approach minimizes experimental variability and accelerates the evaluation of innovative orthodontic configurations.

## 1. Introduction

Significant advancements in dental device modeling and manufacturing have emerged globally, driven by cutting-edge technologies such as 3D scanning and 3D printing. These innovations have revolutionized the creation of dental appliances, orthodontic brackets, and surgical guides [1,2,3]. Furthermore, imaging technologies have progressed to enable 3D internal analyses through X-ray tomography, enhancing diagnostic and treatment precision [4]. This study focuses on the application of finite element method (FEM) simulations in orthodontics, specifically evaluating thermoformed aligners integrated with complementary biomechanical attachments (CBAs).

Currently, it is estimated that between 20 and 30% of the world’s population has alterations in occlusion that require orthodontic treatment [5]. This scenario has motivated the interest of studies aimed to improve the treatment procedures of these clinical conditions [6,7]. For example, according to [8,9], an orthodontic procedure commonly applied to patients with insufficient transverse development of the upper jaw, called a posterior cross bite, is the expansion of the maxillary arch by mechanical devices, such as brackets and thermoformed aligners. Brackets, fabricated from rigid materials, adhere to the tooth surface via dental resins and are interconnected by wires of varying flexibility. These wires are designed to apply specific forces to facilitate desired tooth movements [10]. In contrast, aligners are formed from sheets of thermoformable polymer covering the surfaces of dental crowns [11]. These devices are customized through digital technologies to induce forces to generate the required tooth movement [11]. Some authors suggest the use of aligners in conjunction with complementary biomechanical attachments (CBA), also known as “couplers”, to generate more accurate tooth movements, both in the rotation of the canine and the expansion of the maxillary arch, by orientating the induced forces [12,13]. On the other hand, other authors disagree and suggest that these couplers do not affect tooth retention [14]. This divergence of results suggests that deeper research is required in this area. In this sense, considering the size and variety of injuries at the maxillofacial level, it is of scientific interest to have tools that analyze the different configurations of the devices used in treatments and their effect on the expansion of the maxillary arch.

However, the evaluation of these technologies usually requires in vivo studies and laboratory tests, which may incur operational, practical and ethical problems that hinder the development of a clinical investigation [15]. Additionally, in vivo tests often present inaccuracies when quantifying the behavior of tooth movement and its effects on soft tissue, such as bone or periodontal ligaments (PDLs). That is, it is extremely difficult to faithfully reproduce these behaviors in controlled laboratory tests. In this context, computational modeling emerges as an option in this area, since the finite element method (FEM) can discretely model tooth movement, thereby generating an analytical tool for obtaining approximate solutions to a wide variety of problems at the maxillary level [16].

Dental movement has been widely studied and recognized for its complexity, due to the factors and effects related to the application of a force to the tooth, in conjunction with the interaction models of the bone or periodontal ligaments (PDLs) [12]. Some authors have used FEM to estimate the mechanical responses of biomaterials and tissues. For example, in [15], FEM was used for the analysis of tooth movement considering tissues as homogeneous, isotropic, and non-viscous. Two studies [17,18] presented the responses of PDLs to a load in conjunction with the movement generated by a dental bracket.

In [19], FEM simulations were employed to evaluate the wear at the male–female interface in implant-supported removable prostheses. The results showed that incorporating a flexible shaft, particularly with Nitinol, reduced strain on the female attachment by up to 90%, demonstrating the effectiveness of FEM in prosthetic designs.

In [20], a clinical investigation focused on the evaluation of predictability in tooth movement (a parameter that compares the actual displacement achieved by plaques, with values estimated prior to treatment, using specialized software) when using thermoformed aligners in 64 adult patients. These results showed that the approximations made with FEM models are comparable to reported clinical studies.

In [21], the FEM was used to determine the relationship between the stresses in the PDL with the loads present in the alveolar bone. In [22], the influence of several parameters was evaluated, such as morphology, material properties, and boundary conditions, on the behavior of the PDL and the alveolar bone. In [18], an FEM model was generated to estimate the interaction between the right upper canine, the alveolar bone, and PDL using a thermoformed plastic aligner and two thermoformed CBAs to generate a 0.15 mm distal movement in the aligner.

In [23], numerical research was carried out to estimate the stress distribution in PDLs by thermoformed aligners of different calibers. In [24], an evaluation was performed on the effect of different CBAs on the rotation of the canine teeth; in this case, the computed tomography of a patient was used to define the teeth, PDL and alveolar bone geometry. Although previous studies have used FEM for the analysis of dental movements, according to [25,26], the use of a single mathematical model for the presentation of results can be biased or generate inaccurate results, which suggests further studies need to be performed using FEM in the analyses of dental applications. Additionally, the increased use of thermoformed aligners with CBAs for orthodontic treatments, such as anterior and posterior cross bite, maxillary expansion, and dental crowding, has also been considered [11,17,21,22].

This study contributes to the growing body of knowledge by analyzing the biomechanical effects of CBAs on PDL during transverse maxillary arch expansion, using FEM simulations to provide new insights into this emerging field.

## 2. Materials and Methods

Initially, through a literature review and expert consultation, the physical properties were defined (geometry: thermoformed aligners and complementary attachments), setting the expansion of the maxillary arch, mechanical properties (Young’s modulus, coefficient of friction between the tooth plate and crown), periodontal ligament model and, load state for the required maxillary arch configuration. Subsequently, a CAD model of upper canine, first premolar, second premolar, and first molar teeth were generated showing their respective periodontal ligaments and the configuration of the three different thermoformed plates (without CBA, with CBA in vestibular (VCBA), and by palatal (PCBA)) based on a full-scale cloud of points for all permanent dentition of the patient. Then, the FEM model was generated, which characterized the movement of the expansion of the maxillary arch by specifying the stresses and deformation states in the teeth and periodontal ligament. The model without CBA was compared with a predictability study [20] and the load states [27]. Finally, the results of the models with and without the CBAs were compared to evaluate the effect on the PDL when performing the expansion in the maxillary arch, from the canine to the molar.

In [20], the predictability of arch expansion using thermoformed aligners was investigated. In a sample of sixty-four white adult patients, the mean accuracy of expansion was estimated as 72.8% for the maxilla and 87.7% for the lower arch, a detailed description of the approach performed is described in [28]. According to [29], the sample size was selected by assuming that 70% of the expansion could be achieved with a margin of error of 10%; the calculation was based on a statistical power of 0.8 and a 95% confidence interval. The sample consisted of 41 women and 23 men, with an average age of 31 years. Data were digitized using the Align Technology iTero Element 2^®^ scanner and restoration STL files were generated, both pre- and post-treatment. For 3D inspection and to measure displacement, the software Geomagic Qualify 12.1^®^ form 3D Systems was used. To map out the rehabilitation protocol from the first to the last aligners, Clincheck 6.0^®^ software from Invisalign^®^ was used, taking as a reference the peaks of the teeth, such as the canine, first premolar, second premolar, and first molar. An indicator was established, called the percentage of predictability, which contrasts the displacements achieved by the plaques in patients, compared to what was estimated prior to treatment using the Clincheck^®^ software [28]. This software only considers the displacements or movements of the teeth involved. The predictability percentage is calculated by Equation (1), where, lp, is the predicted length and, lg, is the length achieved; this equation ensures that the calculation does not exceed 100%.

(1) $$\%predc=100\%-\left[(lp-lg)/lg\right]\times 100$$

In this study, four-tooth expansion movement, from canine to molar, were evaluated by applying a 0.15 mm (Δu) vestibular shift in the tooth.

### 2.1. Modeling

#### 2.1.1. Thermoformed Aligners

**Figure 1:**
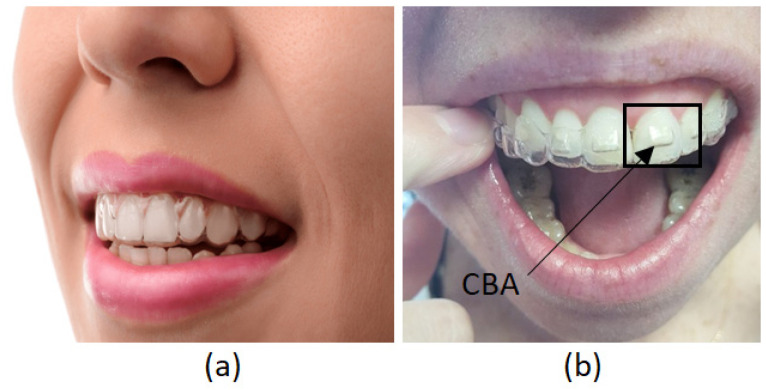
(**a**) Thermoformed plate configuration without CBA. (**b**) Thermoformed plate configuration with CBA.

For this study, one configuration was defined with two conditions, the conditions of the aligner with and without CBAs (see Figure 1a and Figure 1b, respectively). Considering [18], the mechanical properties of the thermoplastic plate were Young’s modulus of 528 MPa and Poisson’s ratio of 0.36.

**Figure 2:**
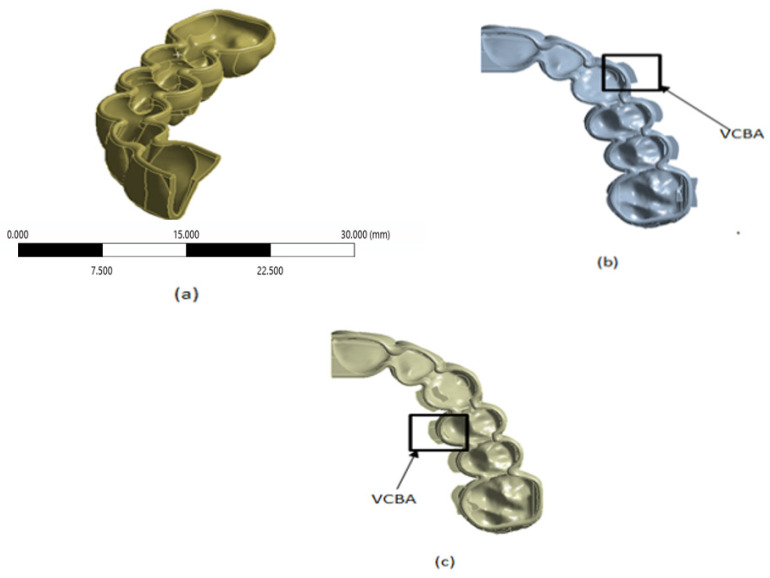
(**a**) Thermoformed plate configuration without CBA. (**b**) Thermoformed plate configuration with vestibular CBA. (**c**) Thermoformed plate configuration with palatal CBA.

Following the method proposed by [22], the thermoformed aligners were modeled using Boolean operations in a 3D parametric CAD program (SolidWorks 2015 version) with a uniform thickness around the crowns of the teeth of 0.7 mm for the following configurations: no CBA, with CBA in the vestibular region, and CBA in the palatal region (Figure 2). In this research, the size, shape, and direction of the attachments were determined by dental orthodontists; this impact on tooth movement will be studied in future work.

#### 2.1.2. Periodontal Ligament (PDL)

The PDL model considered the nonlinear mechanical properties from the experimental data of strength and displacement of the cross sections of the tooth, PDL, and bone. This information was obtained from a 24-year-old male corpse reported in [30].

Shear stress, *τ*, is considered in Equation (2), while shear displacement, γ, is expressed in Equation (3).

(2) τ=force/A s

(3) $$\gamma =\tan^{-1}\left(∆l/W_{L}\right)$$

where As is the cross-sectional area of the tooth root, and PDL; Δl is the elongation obtained in a sample of the PDL after being subjected to at least 3 cycles with a 0.05 MPa load in a material testing machine (MTS 858 Mini Bionix, Eden Prairie, MN, USA); and W_L_ is the thickness of the PDL.

Data were modeled by an exponential function, see Equation (4).

(4) τ=A(eBγ−1)

where parameters A and B were calculated by iterative processes using commercial software reported in [26], obtaining r^2^ > 0.9. Young and Poisson modules were calculated using the same iterative process.

**Figure 3:**
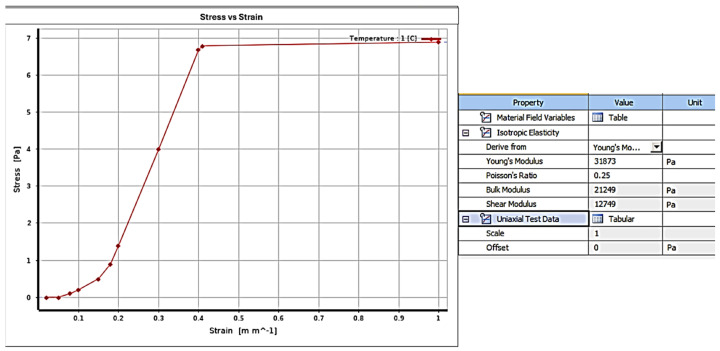
Parameters stress vs. strain curve of PDL in Ansys ®. Source: [18,29].

Based on [24,31], a nonlinear elastic model for PDL was adopted, characterized by the points in Figure 3 which were introduced in Ansys^®^ software v241 with the following parameters: Young’s modulus: 31,873 Pa, Poisson modulus: 0.25, Bulks module: 21,248 Pa, and shear module: 12,749 Pa.

According to [7,17,32], this work simulated the PDL as a uniform thin film of 0.3 mm in thickness around the tooth’s root.

#### 2.1.3. Teeth

To define the material characteristics of the tooth, data from [18,31,33] were used. Young’s modulus: 19.6 GPa, coefficient of Poisson: 0.3, and the linear elastic model were considered. The CAD model of the tooth was generated based on a real scale cloud of points which considered the tri-radicular molar (6), mono-radicular premolar (5), bi-radicular premolar (4), and canine (3), respectively.

#### 2.1.4. Complementary Biomechanical Attachments (CBAs)

**Figure 4:**
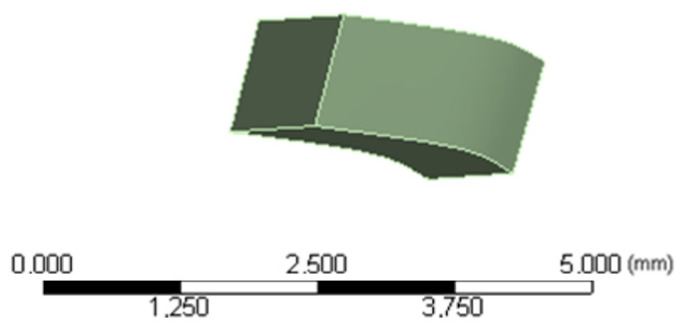
Geometry configuration for CBA. Source: author.

Based on [34], the resin selected was Filtek P60 with Young’s modulus of 1.25 GPa and a Poisson coefficient of 0.36. The CBAs were considered to adhere to half of the crown surface of each tooth. CBA dimensions in canine, the first premolar and second premolar are: length 3 mm, width 1 mm, and deep 1 mm. CBA dimensions in molar are: length 4 mm, width 1 mm, and deep 1 mm. Figure 4 presents canine’s CBA geometry.

#### 2.1.5. Loading and Boundary Condition for FEM Analysis

**Figure 5:**
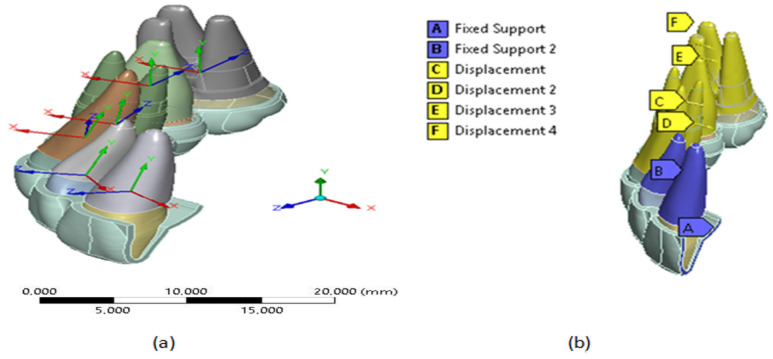
CAD model: (**a**) own coordinate axes on each tooth; (**b**) displacement conditions generated on the outer surfaces of the PDL. Source: author.

To ensure symmetry in the sagittal plane of oral morphology at point (A), see Figure 5; a condition of Fixed Support (displacement = 0) was assumed at the edge of the thermoformed plate [34]. Furthermore, considering that the external surfaces of the PDL are attached to the bone, which has greater structural rigidity, a solidarity movement was assumed through a Fixed Support boundary condition in the region described with the letter A and B, see Figure 5b.

The expansion movement was represented by imposing 0.15 mm shifts towards the vestibular direction (local directions X), as shown in Figure 5a, on the external surfaces of bone–tooth that is in contact with the teeth PDL (canine, first premolar, second premolar, and molar). These displacements are represented by the letters C, D, E, and F, respectively, in Figure 5b. Additionally, they considered the free movement and local addresses fixed Z to ensure displacement in the vestibular direction [35].

**Figure 6:**
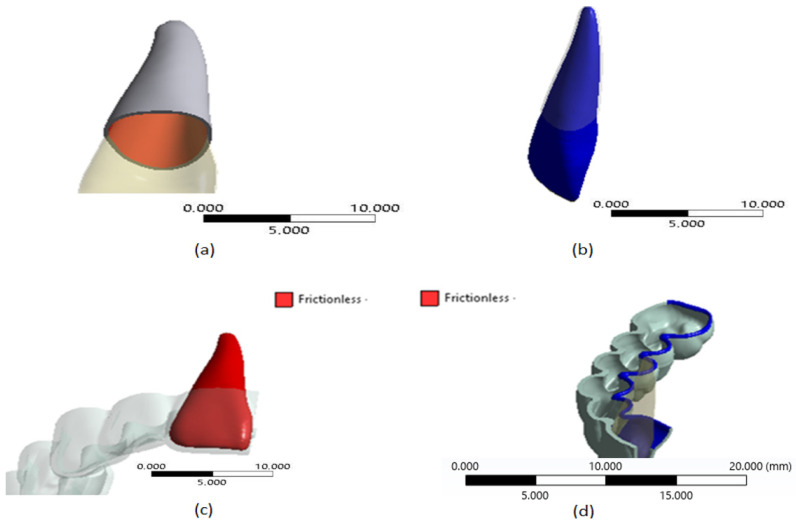
Contact conditions: (**a**) inner surfaces of the PDL, (**b**) tooth surfaces, (**c**) external surfaces of the tooth with plate, and (**d**) surfaces of the plate which are in contact with the tooth. Source: author.

According to [26], it was assumed that the interaction between the contact surfaces of the teeth and their PDL present perfect adhesion; therefore, it was represented by a bonded condition (see Figure 6a,b). Considering that between the polymeric material of the aligner and the biological tissue of the teeth there is the presence of saliva, the interaction between the contact surfaces between the crowns of the teeth and the thermoformed plate was assumed to be a frictionless condition (see Figure 6c,d).

To generate FEM analysis, each outer surface of the PDL was moved 0.15 mm to perform the expansion without considering the effects of bone remodeling. This displacement was assumed in thirty steps, with initial steps of five. The contact stiffness was updated automatically for each iteration. Using the Newton–Raphson method’s iterations, the mismatch criteria were compared to the maximum value of allowed penetration.

Given that this study does not consider bone regeneration, the simulation was carried out in the Ansys’s^®^ Structural static module using an iterative solver. The Newton–Raphson method was used to model the interaction between the mechanical behavior of the PDL and the frictionless contact between the tooth crown and the thermoformed plate.

## 3. Results

The predictability of the model and the length lg were estimated using Equation (4) by comparing the activation distance lp (0.15 mm) prescribed on the contact surfaces between the bone and periodontal ligament.

These results can be observed in Table 1 where the type of tooth, the results obtained by [15,23], and the results obtained in the simulation without CBA were compared.

The results of the FEM model presented an elastic error with reported values of less than 5%.

According to the results of the simulations, the forces exerted on the canine teeth to the molar are less than 0.35 N (35.7 g), according to what is referenced in [36]. The results of the load required for the movement of canine in a mandibular expansion of 0.15 mm in the palatal vestibular direction presented a percentage difference of 17% with those reported in [19]. This difference can be explained since the reference did not consider the effect of the movement of the set of teeth from canine to molar.

**Figure 7:**
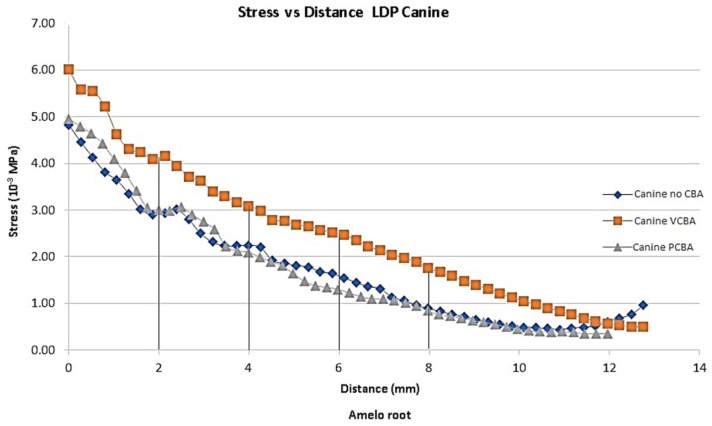
Equivalent stress versus distance canine PDL distance. Source: author.

**Figure 8:**
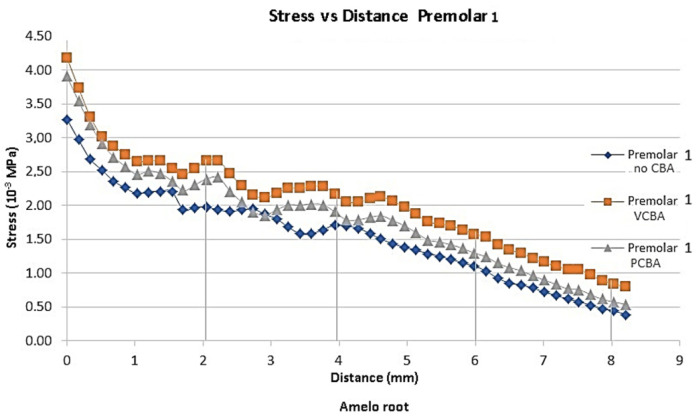
Equivalent stress versus distance first premolar PDL distance. Source: author.

**Figure 9:**
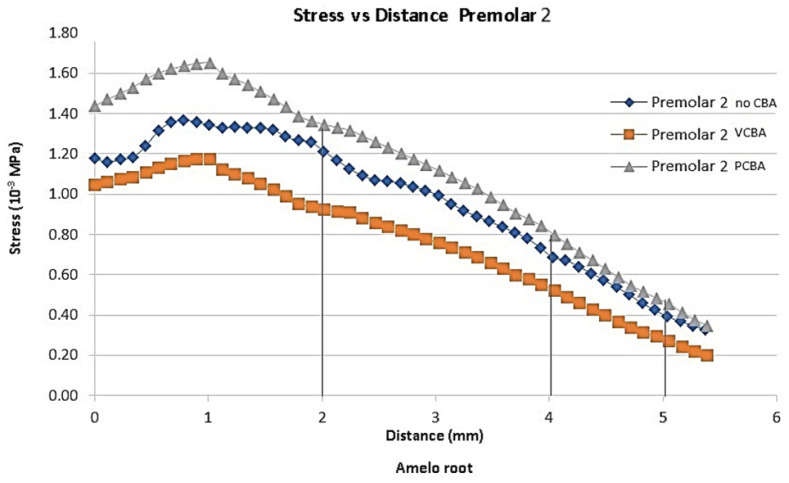
Equivalent stress versus distance second premolar PDL. Source: author.

**Figure 10:**
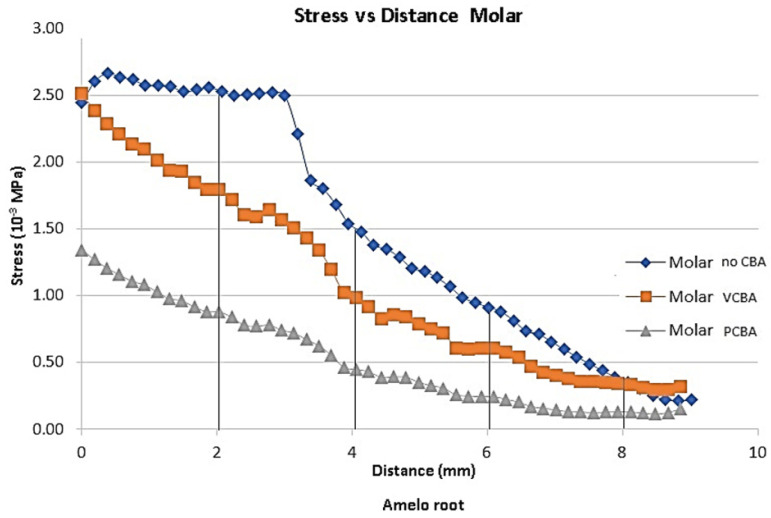
Equivalent stress versus distance molar PDL. Source: author.

In Figure 7, Figure 8, Figure 9 and Figure 10, the variation in the Von Mises equivalent stress is observed along the PDL through the set of teeth in canine, first premolar, second premolar, and molar, considered from the cement–enamel junction (where the PDL begins) to the root.

Reference points were set for canine, first premolar, and second premolar at 2, 4, 6, and 8 mm from the amelocemental junction. For molar, reference points were set at 2, 4, and 5 mm from the amelocemental junction.

Considering the stress distribution, it can be hypothesized that the effects of using CBA exhibit greater relevance based on the separation plane of symmetry. In this sense, second premolar and molar were the ones that presented greater stress values.

In Figure 7, the results for canine shows Von Mises stress of 0.004822 MPa using no-CBA aligners, a stress of 0.004958 MPa using PCBA aligners, and finally 0.006007 MPa using VCBA aligners.

In Figure 8, the results for first premolar show a stress of 0.003273 MPa using no-CBA aligners, a stress of 0.003907 MPa using PCBA aligners, and finally 0.004179 MPa using VCBA aligners. In Figure 12, the results show a load of 0.114 N and a moment of 0.805 N·mm with no CBA. On the other hand, using the PCBA aligner, the load and moment were 0.154 N and 0.942 N·mm, respectively, while the VCBA results show a maximum load and moment of 0.221 N and 0.947 N·mm.

In Figure 9, the results for the second premolar show a stress of 0.001358 MPa at a distance of 1 mm from the amelocemental junction with no need for a CBA aligner. The stress of the VCBA aligner was 0.001173 MPa, while the stress of the PCBA aligner was a maximum of 0.001649 MPA. In this case, the stresses of VCAB are lower than those of PCBA without the need for a CBA aligner in the middle zone.

In Figure 10, the results for molar shows a stress of 0.002520 MPa at a distance of 2 mm from the amelocemental junction with no need for a CBA aligner. The stress of the VCBA aligner was 0.00179 MPa, while the stress of the PCBA aligner was a maximum of 0.0087 MPA. In this case, the stresses of the no-CBA aligner were higher than for VCB and PCBA.

**Figure 11:**
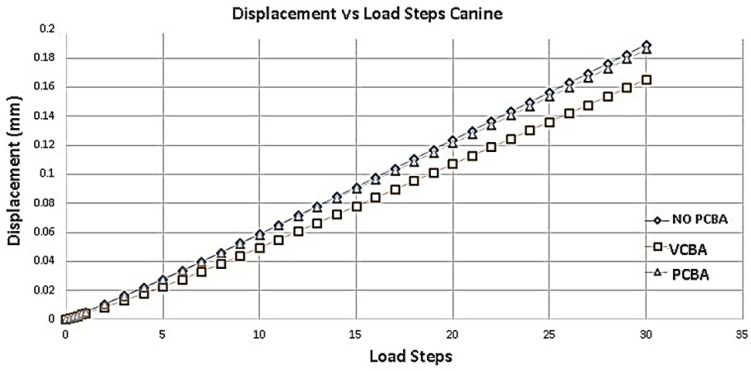
Displacement versus load steps canine PDL. Source: author.

Figure 11 shows the deformation of the PDL of canine with a progressive application of the load. Taking as a reference the results of the deformation of the no-CBA model, a percentage difference of −12.698% with respect to the model with buccal abutments is presented. And, with respect to the model with palatal abutments, the percentage difference is −1.587%. The largest displacements are presented in the no-CBA model.

**Figure 12:**
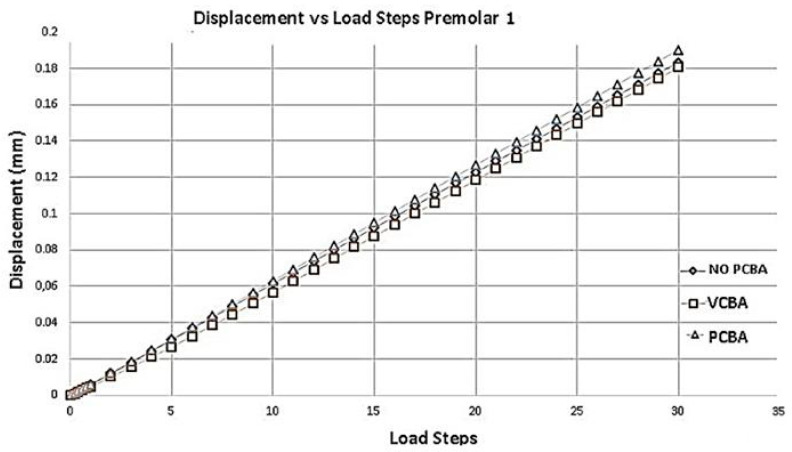
Displacement versus load steps premolar PDL. Source: author.

According to Figure 12, compared to the model with buccal abutments, the deformation of first premolar presents a percentage increase of 1.63%. And, compared to the model with palatal abutments, a percentage increase of 4.91% is presented. The largest displacements are presented in the no-CBA model.

**Figure 13:**
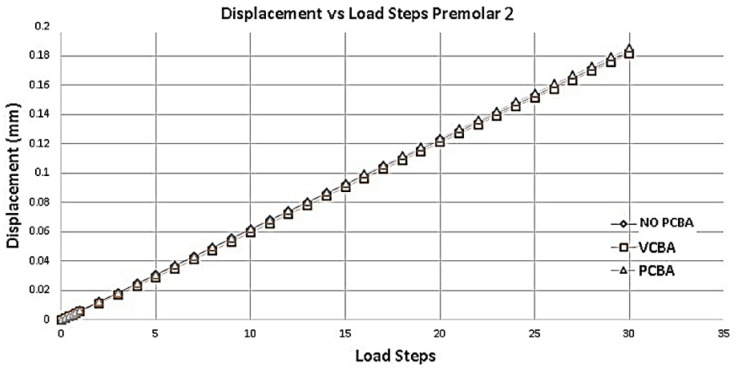
Displacement versus load steps premolar PDL. Source: author.

Figure 13 shows the variation in the total deformation of the PDL of second premolar. Taking the no-CBA model as a reference and compared to the model with buccal attachments, a percentage increase of 1.63% is presented. On the other hand, compared to the model with PCBA abutments percentage increase 4.91%. No CBA presented the largest displacements.

**Figure 14:**
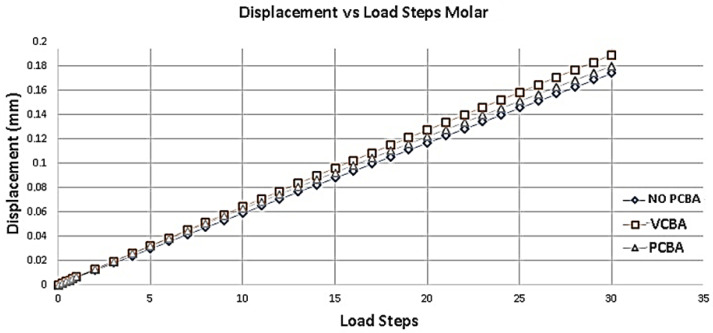
Displacement versus load steps molar PDL. Source: author.

Figure 14 presents the deformation results of molar. Taking as reference the deformation results of the model without CBA, a percentage increase of 7.93% is presented compared to the model with buccal abutments. On the other hand, it presents a percentage increase of 3.17% compared to the model with palatal attachments.

**Figure 15:**
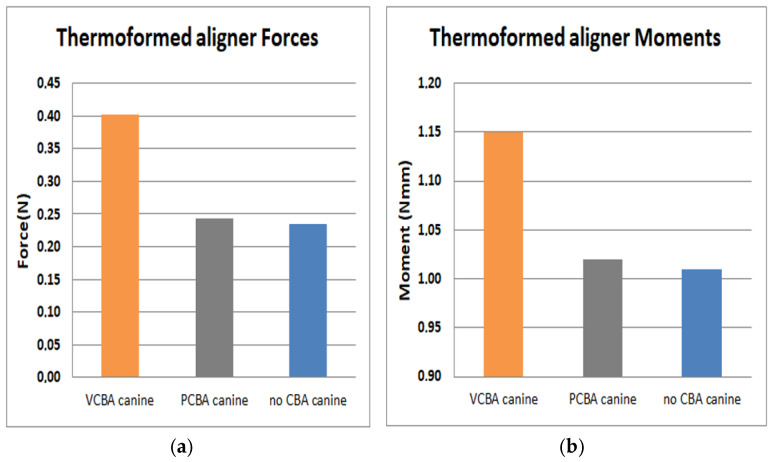
(**a**) Force applied to PDL to move canine. (**b**) Moment applied to PDL to turn canine. Source: author.

**Figure 16:**
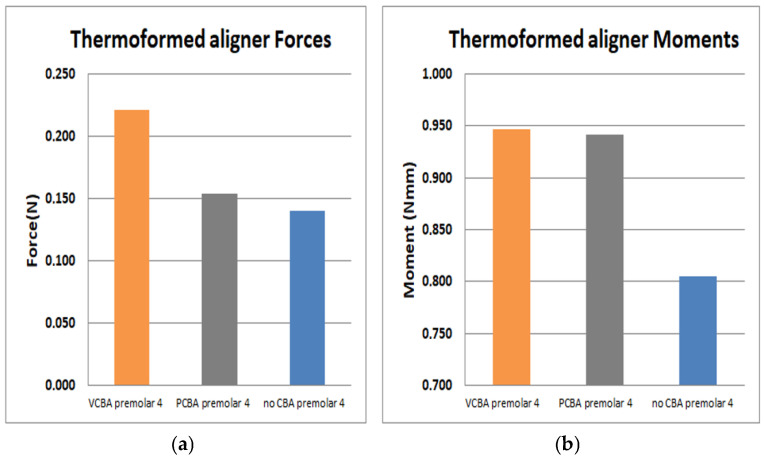
(**a**) Force applied to PDL to move first premolar. (**b**) Moment applied to PDL to turn first premolar. Source: author.

**Figure 17:**
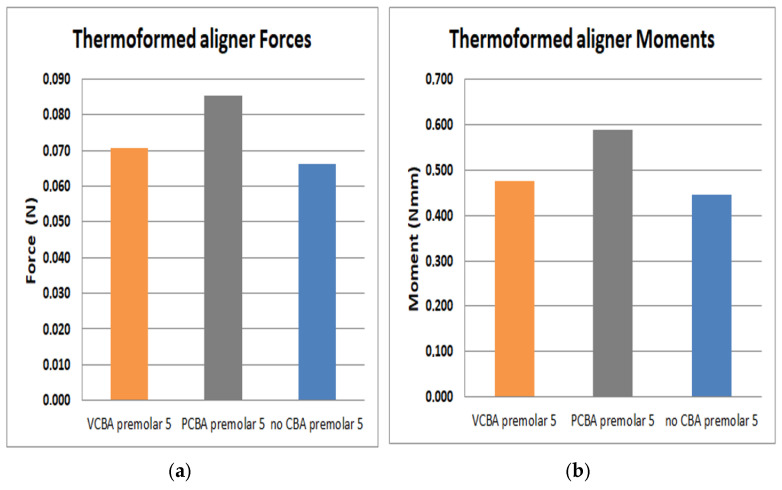
(**a**)Force applied to PDL to move second premolar. (**b**) Moment applied to PDL to turn second premolar. Source: author.

**Figure 18:**
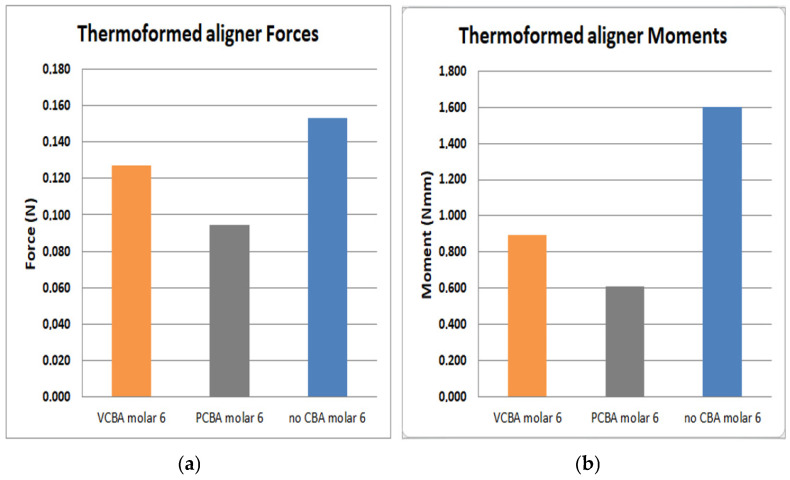
(**a**) Force applied to PDL to move molar. (**b**) Moment applied to PDL to turn molar. Source: author.

In Figure 15, Figure 16, Figure 17 and Figure 18, the forces and moments of canine, first premolar, second premolar, and molar are observed, respectively.

In Figure 15, the results for canine show a load of 0.235 N and a moment of 1.10 N·mm with no CBA. On the other hand, with the PCBA aligner, the load and moment were 0.243 N and 1.02 N·mm, respectively, while the VCBA aligner’s results show a maximum load and moment of 0.402 N and 1.15 N·mm.

In Figure 16, the results for the first premolar show a load of 0.114 N and a moment of 0.805 N·mm with no CBA. On the other hand, using the PCBA aligner, the load and moment were 0.154 N and 0.942 N·mm, respectively, while the VCBA results show a maximum load and moment of 0.221 Nand 0.947 N·mm.

In Figure 17, the results for the second premolar show a load of 0.0661 N and a moment of 0.446 N·mm with no need for CBA. In addition, using a VCBA, the aligner load and moment were 0.077 N and 0.476 N·mm, respectively, while the PCBA aligner results show a maximum load and moment of 0.0854 N and 0.589 N·mm, whilst for second premolar, the forces are lower than canine and first premolar.

In Figure 18, the results for the molar show a load of 0.153 N and a moment of 0.16 N·mm with no need for CBA. In addition, using the VCBA aligner the load and moment were 0.127 N and 0.896 N·mm, respectively, while the PCBA aligner results show a maximum load and moment of 0.0946 N and 0.607 N·mm. In this case, the forces and moments are higher using a no-CBA aligner.

In the FEM simulation, it was evidenced that the tipping effect (combined translation and rotation of the tooth) was lower with CBAs, so whilst there were higher forces and moments, tipping was reduced.

## 4. Discussion

This study enhances the estimation of tooth movement in orthodontics by providing a predictive and highly accurate framework for analyzing the forces and displacements generated by different aligner systems. The numerical models presented enable the precise quantification of force systems acting on teeth when clear aligners are used in combination with various types of composite attachments (CBA, VCA, and PCBA). These simulations offer critical insights into the interactions among aligners, the periodontal ligament (PDL), and surrounding bone structures.

Furthermore, these models elucidate how the shape and positioning of attachments influence the effectiveness of aligners in achieving controlled tooth movement. Visualization of motion and stress distribution, particularly with CBA, VCBA, and PCBA, allows clinicians to make informed decisions regarding treatment approaches based on the predicted displacement patterns observed in the simulations. Overall, this study underscores the significance of computational modeling as an essential tool in modern orthodontics. By improving treatment predictability, enhancing patient-specific customization, and optimizing aligner design for more efficient force application, these findings contribute to the advancement of precision-driven orthodontic treatments.

**Figure 19:**
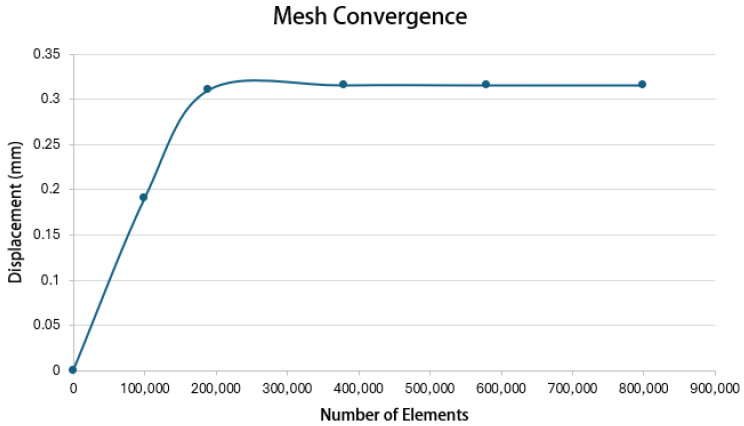
Mesh convergence in displacement simulation of canine. Source: author.

In this FEM model, isotropy is assumed for the mechanical properties of periodontal ligament (PDL) and alveolar bone [37]. This simplification can lead to deviations between simulated and actual tissue responses. In addition, defining appropriate boundary conditions and loading scenarios is critical for the FEM analyses; therefore, inaccuracies or oversimplifications in these parameters can lead to results that do not accurately reflect clinical conditions. The reliability of the FEM model used in this study is based on mesh convergence, with a 5% deviation criterion; Figure 19 shows the mesh convergence for the canine simulation.

Comparisons between the predictability percentage from a clinical case [38] and FEM simulations produced values of 1.13% for canine; 2.01% for first premolar; 2.45% for second premolar; and 3.0% for molar. Detailed results are as follows:

The largest displacements were presented in the non-CBA model, with little difference with respect to the PCBA model. Additionally, the movement of maxillofacial expansion influences the results of the VCBA model.

For canine, the variation percentages between VCBA and no-CBA stress were 42%, 35%, 60%, and 102% from the reference points. The variation percentages between PCBA and no CBA were 2%, −10%, −21%, and −8%, respectively. These variations highlight that the VCBA aligner generates higher stress.

For the first premolar, variation percentages of stress with VCBA and no CBA for premolar 4 were 35%, 21%, 43%, and 95%, respectively, from the reference points. The PCBA variations were 21%, 5%, 12%, and 33%. This concludes that VCBA generates higher stress than PCBA aligners.

For the second premolar, the stress VBCA variations were −24%, −25%, and −36%, and for PCBA variations, they were 11%, 16%, and 15%. The variation values underline that VCBA generates less stress than no CBA aligners.

For the first molar using VCBA and PCBA aligners, the stresses were lower than for no CBA, and the variation percentages for VCBA were 229%, −33%, −34%, and −3%. The variation percentages for PCBA were −65%, −70%, −74%, and −65%.

It was found that the forces and moments in canine were maximum with VCBA aligners, producing values of 0.402 N and 1.15 N·mm. For first premolar, a maximum force and moment of 0.221 N and 0.947 N·mm were generated with the VCBA aligner. Additionally, for second premolar, the maximum force and moment were obtained using the PCBA aligner with values of 0.854 N and 0.589 N·mm, respectively. Finally, in molar, the maximum forces and moments produced were 0.153 N and 1.6 N·mm, with no CBA.

On the other hand, bone density varies significantly among individuals and can impact the forces required for effective tooth movement. Integrating patient-specific bone density data into FEM models would allow for more precise predictions and better alignment of treatment strategies with individual patient needs; however, bone density data studies are outside the scope of this study.

## 5. Conclusions

Simulations have shown that the use of CBA reduces tipping in teeth, and the model with VCBA presented the best results for dental movement.

Considering the practical and ethical problems of clinically judging the differences between CBA and no CBA expansions with aligners, the computational model is an approach that can be used to estimate tooth movement. In addition, the resulting forces and moments acting on the dental system imposing distal displacement on aligners could be calculated. In fact, the simulations showed that a greater reaction, represented as an increase in force and moment, presents lower tipping. In this case, the use of CBA reduces tipping in teeth, and the model with VCBA presented the best results for dental movement.

The results indicate that the presence or absence of CBA influences the magnitude of relative tooth movement, which varies depending on the tooth’s position within the treatment plan, but the use of CBA (vestibular o palatal) or no CBA depends on the expertise of the oral medicine professional, who must determine the appropriate type of movement required for a specific treatment.

## Figures and Tables

**Table 1:** Comparation between clinical outcome and simulation results without cba.

Tooth	% Change Accuracy (Clinical Outcome) [15,23]	% Change Accuracy (Simulation Result)	Relative Error
Canine	88.7	89.7	1.13%
First premolar	84.7	86.4	2.01%
Second premolar	81.7	83.7	2.45%
Molar	76.7	79	3.00%

## Data Availability

Restrictions apply to the datasets: the datasets presented in this article are not available because the data are part of a different studies with data access restrictions.
